# Analysis of the effect of neuroendoscopy-assisted microscopy in the treatment of Large (Koos grade IV) vestibular schwannoma

**DOI:** 10.3389/fonc.2023.1033954

**Published:** 2023-01-17

**Authors:** Zhenxing Yang, Xiaoxing Xiong, Zhihong Jian, Li Du

**Affiliations:** ^1^ Department of Neurosurgery, Renmin Hospital of Wuhan University, Wuhan, Hubei, China; ^2^ Department of Anesthesiology, Renmin Hospital of Wuhan University, Wuhan, Hubei, China

**Keywords:** vestibular schwannoma, neuroendoscopy, internal auditory canal, facial nerve protection, hearing preservation

## Abstract

**Introduction:**

This article aimed to investigate the effects of the endoscopic-assisted microsurgery technique on the resection of large (Koos grade IV) vestibular schwannoma (VS) and provide a prognosis analysis of the patients.

**Methods:**

A retrospective analysis of the use of the endoscopic-assisted microsurgery technique in 16 cases of large vestibular schwannoma surgery was carried out. Intraoperative nerve electrophysiological monitoring was conducted to explore the effect of neuroendoscopy on the resection of internal auditory canal tumors, protection of the facial nerve, and minimizing postoperative complications.

**Results:**

Tumors were completely removed in all 16 cases, and the facial nerve was anatomically preserved in 14 cases (87.5%). There was no postoperative cerebrospinal fluid leakage and no intracranial infection complications occurred.Following the House-Brackmann (H-B) grading system, post-operative facial nerve function was grade I in 5 cases, grade II in 6 cases, grade III in 3 cases, and grade V in 2 cases. As a result, the preservation rate of facial nerve function (H-B grade I-II) was 68.8%. All 16 patients were followed up for 3 to 24 months, and no tumor recurrence was found on enhanced MRI.

**Discussion:**

Using the endoscopic-assisted microsurgery technique in the retrosigmoid approach has many advantages over the microscopic-only approach. When compared to the microscopy-only approach, the endoscope can provide a wide-angle surgical field superior to that of a microscope in areas such as the internal auditory canal in the resection of large VS, minimize iatrogenic injuries, ensure complete removal of internal auditory canal tumors, and well as reducing postoperative complications such as cerebrospinal fluid leakage and the loss of facial and auditory nerve functions.

## 1 Introduction

Vestibular schwannomas (VS) are benign neoplasms arising from Schwann cells of the vestibulocochlear nerve (1), mostly from the vestibular part of the internal auditory canal. 75% of VS originate from the superior vestibular nerve, while the rest 25% originate from the cochlea. Accounting for about 90% of adult cerebellopontine angle tumors and 8% of intracranial tumors, VS is the most common benign tumor in the internal auditory canal and cerebellopontine angle (2). At present, more emphasis is being placed on total tumor resection, intraoperative nerve function preservation (facial and auditory nerves), postoperative complications reduction, and overall quality of life improvement using endoscopic-assisted techniques (3, 4). Due to the adult central neurons’ lack of regenerative capacity and the irreversible nature of any cochlear and facial nerve damage sustained during acoustic neuroma removal, permanent facial paralysis and hearing loss result (5). In this article, we retrospectively analyzed the surgical outcomes of 16 cases of large vestibular schwannomas treated with endoscopic-assisted microsurgery *via* a retrosigmoid approach at Wuhan University’s Renmin Hospital from May 2019 to July 2022. The report is as follows.

## 2 Methods

### 2.1 Patient and data collection

Electronic medical records were reviewed for patient demographic information, treatment, and audiometric data. The inclusion criteria for this study were: (1) an enhanced MRI scan diagnosed Vestibular schwannomas with a maximum diameter of more than 3 cm and tumor invasion into the internal auditory canal (Koos grade IV); (2) received operation treatment at the neurosurgery department; (3) a postoperative pathological examination confirmed Vestibular schwannomas. Patients who did not meet the above criteria were excluded from this study. The gender, age, clinical symptoms, hearing examination results, and facial nerve function evaluation results before and after surgery of patients meeting the above conditions were retrospectively analyzed.

### 2.2 Imaging and hearing examination

All 16 cases underwent a preoperative thin-slice CT scan of the skull base and a 3.0T MRI with gadolinium. Brainstem Auditory Evoked Potentials (BAEP) and pure tone threshold test dates were collected for these patients before the operation. 3 months after the operation, all the patients returned to our department for a Thin-slice CT scan of the skull base and 3.0T MRI with gadolinium, as well as a facial nerve function examination and hearing test. Follow-up inspection should be conducted every six months.

### 2.3 Surgical technique

This is a single-surgeon retrospective series in which all cases were performed by the same senior surgeon and assistants. After successful intubation under general anesthesia, the patient is placed in the park-bench position for a retrosigmoid approach. Compared with the semi-sitting or Jannetta position, this position is easy to place, and the surgery is more likely to expose tumors in the CPA area, which is convenient for our subsequent combined operation of microscope and neuroendoscope. Therefore, we routinely choose this surgical approach, the head is fixed by the frame, the root of the mastoid is at the highest point, the shoulder is retracted downward, and an arc incision is made behind the suboccipital sigmoid sinus behind the ear on the diseased side, up to the upper nuchal line 1.5 cm down to 2 cm below the level of the mastoid tip. Routinely sterilized drape, the skin and subcutaneous tissue are incised layer by layer, 125 ml of 20% mannitol is then rapidly infused intravenously to reduce intracranial pressure, drilled with an electric drill and the bone flap is milled off, the occipital dura is pulled downward to the skull base further exposing the area in front of the midline of the sigmoid sinus, up the transverse sinus, down to the lateral border of the foramen magnum. The bone window is about 4*3cm in size.

Microscopic resection of tumors in the cerebellopontine angle: German STORZ rigid endoscope was used during the operation, the rigid endoscope was 17 cm in length, 4.0 mm in diameter, and the angle was 30°. The endoscope was fixed with a Snake brand mechanical fixed arm, which could be adjusted in depth and angle at any time as needed. Under the microscope, the dura mater was cut along the sinus edge in an arc and retracted from the sinus edge to fully expose the cerebellopontine angle after releasing the cerebrospinal fluid (CSF) from the cisterna magna. The relationship between the tumor and the surrounding structures such as the trigeminal nerve, posterior cranial nerve, and blood vessels was first observed under the microscope, and the facial nerve monitoring response was observed by stimulating the tumor surface to confirm the location of the facial nerve. Then, the two layers of the arachnoid membrane along the tumor surface were separated under the microscope, the tumor capsule was cauterized and the tumor capsule was excised in pieces. When the tumor in the cerebellopontine angle has been separated to the deep part of the tumor after subtotal resection and decompression, a 30° endoscope is placed, the residual tumor and the brainstem surface, and the surrounding trigeminal nerve, trochlear nerve, petrosal vein, and supra cerebellum are carefully observed from multiple angles. The tumor capsule wall is peeled from the brainstem surface under the endoscope to avoid excessive stretching of the cerebellum and brainstem, and then the tumor and the facial nerve are carefully removed from the internal auditory canal opening. Attention should be paid to the principle of bidirectional separation. Stimulator stimulation helped identify the location of the facial nerve. After the tumor was removed at the cerebellopontine angle, the wound was covered with a gelatin sponge to protect the latter group’s cranial nerves and blood vessels.

Treatment of residual tumors in the internal auditory canal: The lip of the internal auditory canal is where the tumor most closely adheres to the dura mater. The posterior lip of the internal auditory canal is removed with a small emery drill. Pay attention to continuous flushing and grinding at low speed to avoid excessive local temperature. When cauterizing the nerve in the internal auditory canal, the width and depth of the posterior wall should be enough to fully expose the tumor in the internal auditory canal, generally 5-6 mm. After incising the dura covering the internal auditory canal with a hook knife, a 30° neuroendoscope was placed again to observe the relationship between the residual tumor and the nerve in the internal auditory canal. Almost always avoid blindly grasping and pulling the tumor with forceps. After the internal auditory canal tumor is completely removed, the facial, vestibular, and cochlear nerves can be distinguished under endoscopy, and the facial nerve is stimulated again to observe its function. The petrosal bone on the posterior wall of the internal auditory canal was examined to check whether the mastoid air cell was open, sealed with bone wax, packed with muscle fragments, and then fixed with biological protein glue to avoid leakage of CSF. After carefully achieving the hemostasis of the surgical field, the dura mater was sutured, the bone flap was fixed, and the muscle and skin were sutured in layers.

## 3 Result

Our review included 16 patients, 9 males, and 7 females; the age ranged from 33 - 75 years old, and the medical history was from 2 months to 15 years. There were 13 cases of tinnitus and partial hearing loss, 3 cases of complete hearing loss, 9 cases of dizziness, and 3 cases of limb ataxia; preoperative facial nerve function was classified according to House-Brackmann (6), as grade I in 8 cases, II in 6, III in 1 and IV in 1. hearing loss according to the WHO classification (7), as mild hearing loss in 2 cases, moderate hearing loss 3, moderately severe hearing loss 4, severe hearing loss 2, profound hearing loss 2, complete hearing loss 3 cases, respectively (**Table 1**). The tumor size (longest diameter of axial plane) was between 36 and 62 mm (average 43.6 mm). According to the Koos classification (8), all tumors were grade IV. The results of Brainstem Auditory Evoked Potentials (BAEP) and pure tone threshold test showed decreased hearing of varying degrees on the affected side, among which 3 cases had complete hearing loss.

**Table 1:** Clinical characters of patients.

No	Gender	Age	Ear	Main Symptoms	Max Diameter mm	Toumor Size Koos Grade	Facial Function (HB-grade)	Hearing Lose Grade
1	M	48	L	Tinnitus 5ys, HL 3ys	45	IV	I	MS
2	F	36	L	Vertigo with tinnitus 6ms	42	IV	I	Mild
3	F	52	R	Tinnitus 6ys, HL 3ys	46	IV	II	Severe
4	M	64	L	HL 3ys Vertigo 5ms	38	IV	II	Moderate
5	F	75	R	Deaf 15ys Ataxia 6ms	62	IV	II	deafness
6	F	56	R	Vertigo 8ms	38	IV	I	Moderate
7	M	46	L	HL 2ys	44	IV	II	MS
8	M	54	R	Deaf 3ys hoarseness 6ms	48	IV	III	deafness
9	M	62	R	Deaf 5ys FP 2 ms	49	IV	IV	deafness
10	F	33	L	Tinnitus 2ys	36	IV	I	Mild
11	F	70	R	HL5ys	52	IV	II	Profound
12	M	62	R	HL Tinnitus 3ys	38	IV	I	MS
13	M	58	L	Vertigo Ataxia 2 vs,	40	IV	II	Profound
14	F	49	R	HL2ys	44	IV	I	Moderate
15	M	51	L	HL 2ys Tinnitus 6ms	39	IV	I	MS
16	M	57	L	HL 3ys Ataxia 2 ms	54	IV	I	Severe

Maximum diameter of the tumor was measured from axial contrast-enhanced scan of the MRI; HL, hearing loss; ys, years; ms, months; FP, facial paralysis; MS moderately severe hearing lose.

**Figure 1:**
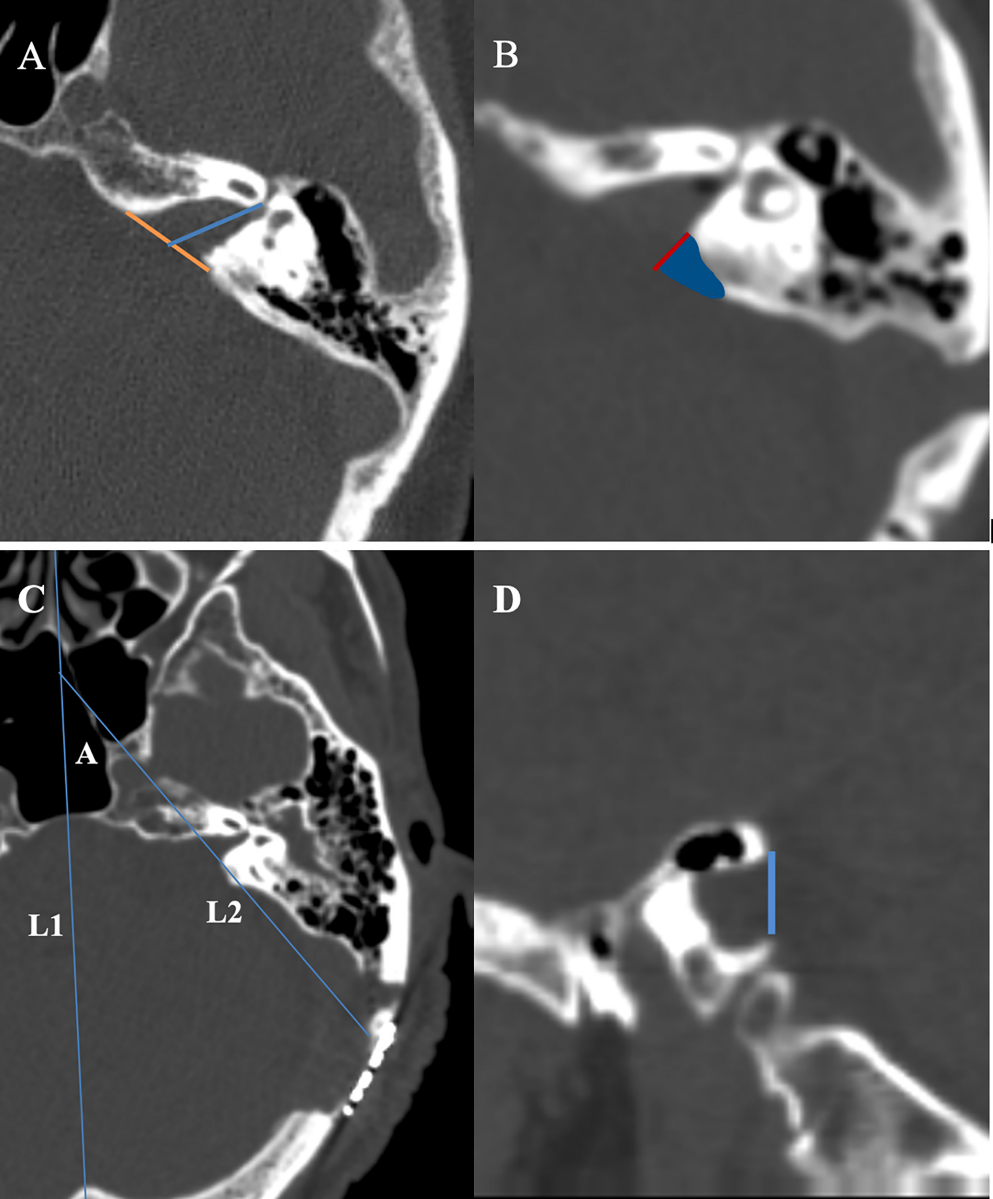
**(A)** Measurement of the absolute tumor extension into the internal auditory canal. On a thin slice CT scan, the slice with the most lateral extension of the tumor is selected for measurement. An auxiliary line (yellow) is drawn between the anterior and posterior lip of the porus acoustics. The length of absolute tumor extension is defined as the distance between the midpoint of the yellow line and the most lateral point of tumor extension. **(B)** We use Brainlab software to fuse the thin-slice CT scan images before and after surgery. The blue area shows the bone removal part of the internal auditory canal, and the length of the red line represents the exposure length of the internal auditory canal. **(C)** L1: the middle sagittal line at the axial plane; L2:the line of the bone removal plane of the posterior and lateral wall of the internal auditory canal at the axial plane; angle A the angle between L1 and L2 stands for the drilling angle. **(D)** The width of internal auditory canal exposure is defined as the distance as a blue line at the sagittal plane.

**Figure 2:**
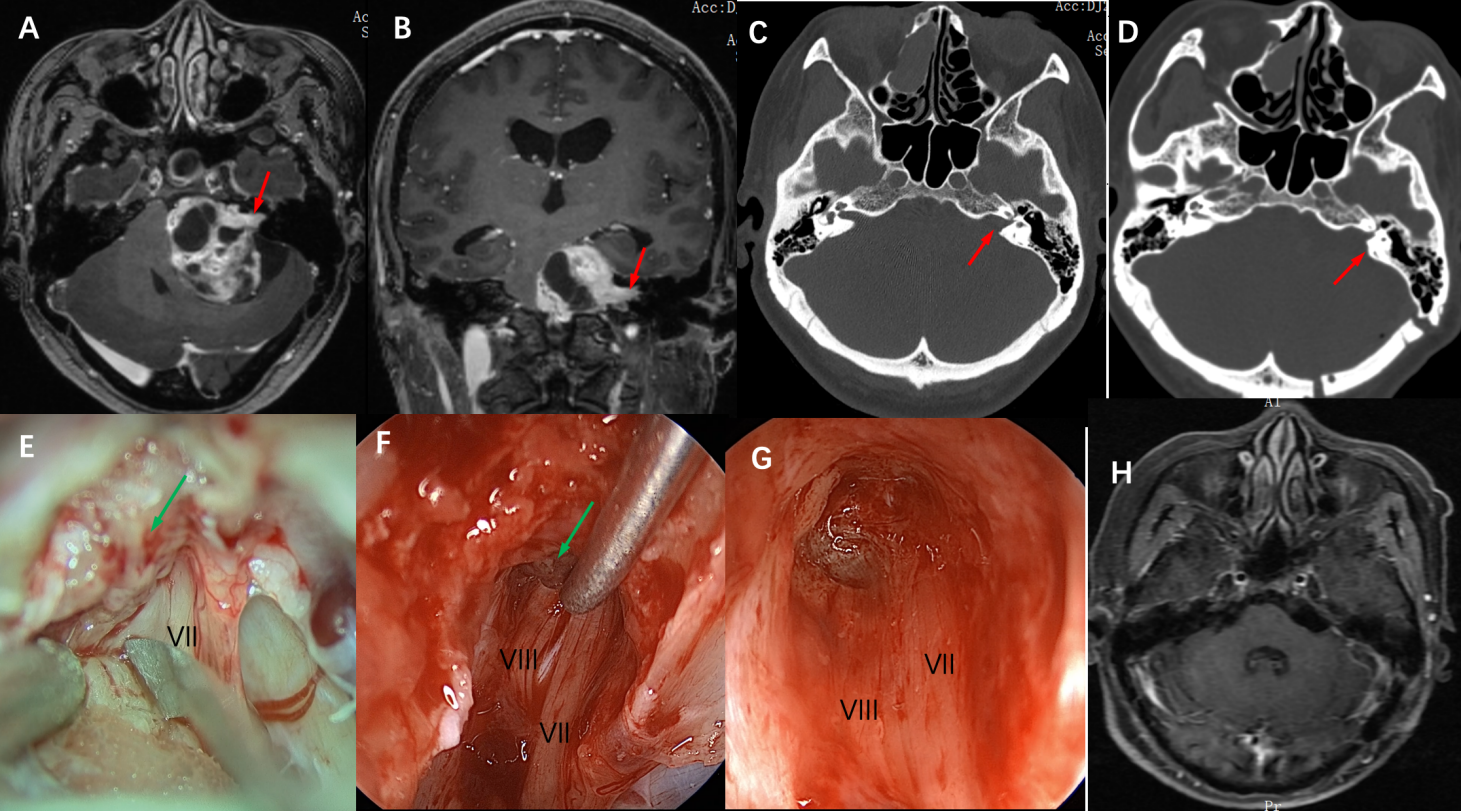
Imaging studies and intraoperative snapshots of a case of VS with an irregularly shaped tumor extending into the internal auditory canal. **(A)** The preoperative T1 MRI with contrast image revealed a giant vestibular schwannoma abutting the brainstem. The intracanalicular portion of the tumor was irregularly shaped as indicated by the red arrow (axial plane). **(B)** giant vestibular schwannoma abutting the brainstem and extension into the internal auditory canal as indicated by the red arrow (coronal plane) **(C)** Thin-section CT scan of the skull base showed that the ipsilateral internal auditory canal was significantly enlarged compared with the contralateral side (red arrow), and the mastoid air cells were well gasified. **(D)** A postoperative CT scan showed that the posterolateral part of the internal auditory canal bone was removed (red arrow), and the structure of the bony labyrinth and semicircular canal was intact. **(E)** Intra-operative microscopic view of the operation showed that after the tumor was removed in the CPA area, the internal auditory canal was opened and there were still a large number of tumor residues in the internal auditory canal (indicated by green arrow), and the VII nerve was located anterior and inferior to the internal auditory canal. **(F)** Intraoperative neuroendoscopy view showed tumor resection in the internal auditory canal, and the tumor was separated from the VII, and VIII nerves using a microscopic ball-tip dissection (green arrow). **(G)** Intraoperative neuroendoscopy view showed that after complete resection of the tumor in the internal auditory canal, the bottom structure of the internal auditory canal showed, and the VII and VIII nerves were intact. **(H)** Postoperative MRI review after 6 months showed complete tumor resection and no tumor recurrence.

**Figure 3:**
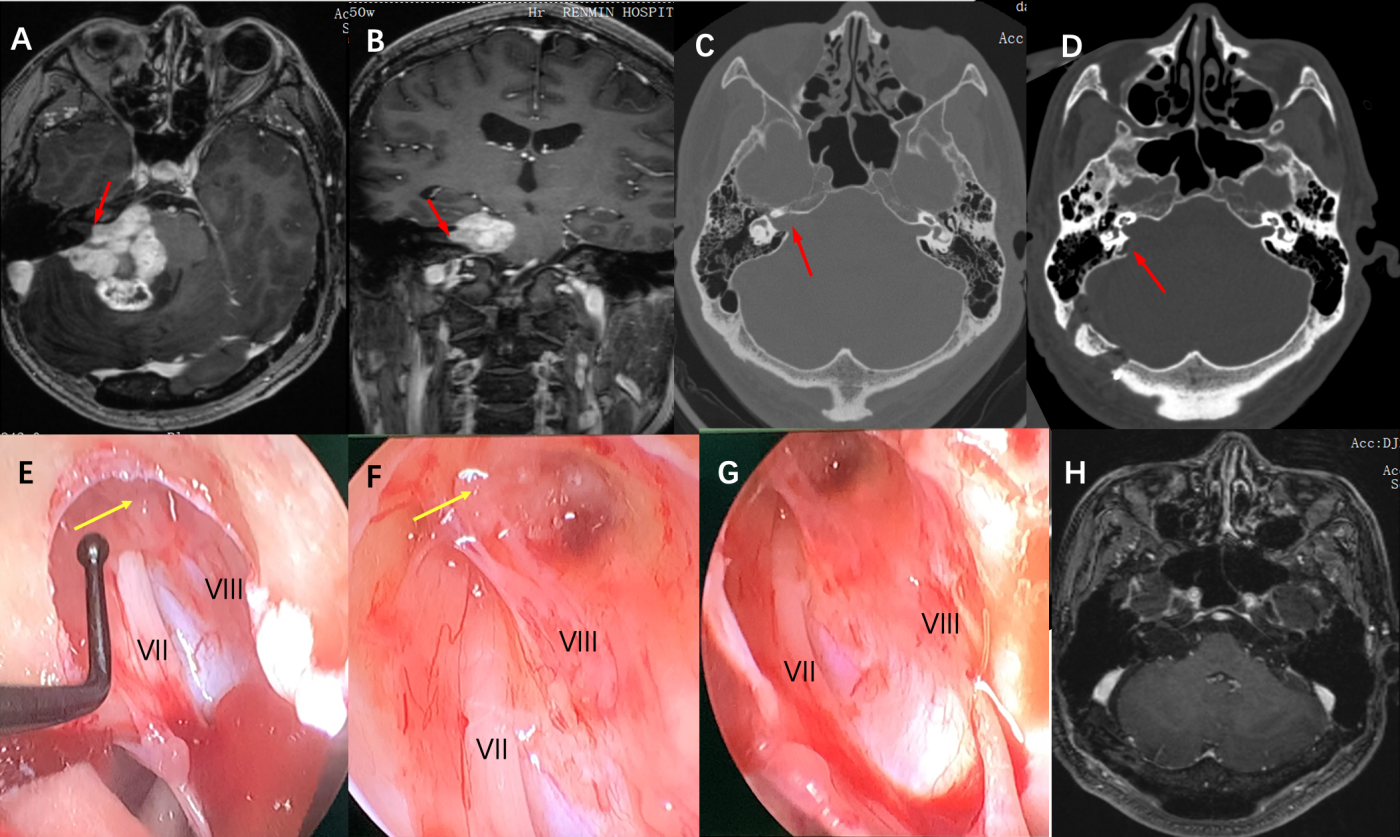
Imaging studies and intraoperative snapshots of a case of Grade IV VS extending into the internal auditory canal. **(A)** The preoperative T1 contrast image revealed a giant VS at the CPA area. The intracanalicular portion of the tumor was irregularly shaped as indicated by the red arrow (axial plane). **(B)** giant VA abutting the brainstem and extension into the internal auditory canal as indicated by the red arrow (coronal plane) **(C)** Thin-section CT scan of the skull base showed that the ipsilateral internal auditory canal was significantly enlarged compared with the contralateral side (red arrow), and the mastoid air cells were well gasified. **(D)** A postoperative CT scan showed that the posterolateral part of the internal auditory canal bone was removed (red arrow), and the structure of the bony labyrinth and semicircular canal was intact. **(E)** Intra-operative neuroendoscopy view of the operation showed that after the tumor was removed in the CPA area, the internal auditory canal was opened and there were tumor remnants in the internal auditory canal (yellow arrow), and nerves VII and VIII are located anterior and posterior inferior to the internal auditory canal respectively. **(F)** Intraoperative neuroendoscopy view showed after tumor resection, there were still tumor remnants at the bottom of the internal auditory canal adhering to the transverse crest (yellow arrow). **(G)** Intraoperative neuroendoscopy showed that after complete resection of the tumor in the internal auditory canal, the bottom structure of the internal auditory canal showed, and the VII and VIII nerves were intact. **(H)** Postoperative MRI review after 1 year showed complete tumor resection and no tumor recurrence.

Tumor tissue was collected from the 16 cases and sent to pathology for examination, and all of them were confirmed to be vestibular schwannoma by immunohistochemical staining. 16 cases achieved gross total resection, and the facial nerve was anatomically preserved in 14 cases (87.5%), The average length of bone resection of the internal auditory canal was 5.5 ± 0.5mm during the operation contrast the total length of the internal auditory canal was 10.7 ± 0.6mm (**Figure 1**). 5 cases of grade I, 6 cases of grade II, 3 cases of grade III, and 2 cases of grade V. 1 patient had developed delayed hemorrhage, but due to the small amount of bleeding, the hematoma was absorbed with conservative management, and was then discharged after recovery. As for postoperative hearing function, 11 patients had varying degrees of hearing loss, and 6 patients had complete hearing loss. There was no CSF leakage, wound infection, or hydrocephalus complications in this group of patients postoperatively. (**Table 2**). The 16 patients were followed up for 3 to 24 months after the operation, and no tumor recurrence was observed during the follow-up (**Figure 2**, **3**).

**Table 2:** Surgical date and follow-up of the patients.

NO	LCA Length (mm)	LCA EL (mm)	Tumor Resection	Follow-up Time	FNF (HB-grade)	HF	Change of HF	Complication	Tumor Recurrence
1	12	5.6	TR	1 year	I	severe	↓	No	No
2	11.4	5.8	TR	6 months	II	MS	↓	No	No
3	12.3	6.2	TR	2 years	III	profound	↓	No	No
4	10.2	5.4	TR	2 years	II	severe	↓	No	No
5	11	5.3	TR	6 months	V	deafness	=	No	No
6	9.2	6	TR	1 year	I	severe	↓	No	No
7	10.3	5.8	TR	1 year	III	deafness	↓	No	No
8	9.4	6.4	TR	3 months	III	deafness	=	No	No
9	11	5.4	TR	1 year	II	deafness	=	No	No
10	9.8	4.8	TR	6 months	I	mild	=	No	No
11	10.2	6.2	TR	2 years	II	severe	=	delayed hematoma	No
12	9.5	5.2	TR	1 year	II	severe	↓	No	No
13	10.6	4.5	TR	6 months	V	deafness	↓	No	No
14	10.2	5.4	TR	3 months	I	MS	↓	No	No
15	11.2	4.8	TR	1 year	I	severe	↓	No	No
16	12.4	5.2	TR	2 years	II	deafness	↓	No	No

TR, Total resection; LCA EL, ICA Exposure Length; FNF, facial nerve function; HF, hearing function; CHF, Change of Hearing Function; ↓, Hearing Function worse than preoperation; = Hearing Function have no change compare to preoperation.

## 4 Discussion

Large VS are often closely adhered to the brain stem, facial nerve, glossopharyngeal nerve, vagus nerve, and cranial roots of the accessory nerve, they are closely related to the anterior inferior cerebellum, posterior inferior arteries, and petrosal veins. Variant degrees of hearing loss, facial nerve paralysis, brainstem injury, or even death are expected when such structures are damaged (9). An increase in tumor size can increase postoperative complications, due to the formation of more adhesion bands between tumor mass and other adjacent structures such as the brainstem, thus patients are at a higher risk of developing respiratory failure within 24 hours postoperatively (9). At present, an endoscopic-assisted technique for more complex cases of VS has been adopted widely by increasing numbers of neurosurgeons, due to the technological advancement of endoscopic-assisted microsurgery and improved intraoperative neurophysiological monitoring technology (10, 11). Endoscopic-assisted microsurgery has greatly reduced postoperative complications, significantly improved the tumor resection rate, and notably has improved the quality of life of patients (12, 13).

Using an endoscopic-assisted technique in internal auditory canal tumor resection can significantly reduce damage to the adjacent nerves and the residual internal auditory canal tumor, thus decreasing the chances of tumor recurrence (14–16). The vast majority of acoustic neuromas originate from the vestibular nerve in the internal auditory canal and then grow outward into the cerebellopontine angle. Recurrence of acoustic neuromas is highly related to residual tumors in the internal auditory canal (12, 13). McKennan et al. were the first to use an endoscopic-assisted technique to explore the internal auditory canal, removing the portion of the VS in the internal auditory canal with preservation of facial nerve (17). Valtonenet al reported that in 78 cases of VS patients endoscope was permitted to identify and remove tumor residues at the bottom of the internal auditory canal in 11 (18). Other retrospective studies reported that the anatomical preservation rate of the facial nerve in VS was 73% to 93% (14–16). However, the anatomical preservation of the facial nerve is not an indicator of the integrity of the facial nerve function. Sobieski reported that a preservation rate of facial nerve function (H-B grade I to II) in of 53.4%, but for tumors with a diameter greater than 3 cm only 35.7% (15). Zhao et al. (19) reported that 33 cases of acoustic neuroma with a diameter of more than 3 cm had a facial nerve function preservation rate of 27.5%. Springborg (14) retrospectively analyzed 1244 cases of acoustic neuroma after surgery and found that the preservation rate of facial nerve function was only 55.6% with a tumor diameter greater than 25 mm. The anatomical preservation rate of our group of 16 patients was 87.5%, with a rate of 68.8% facial nerve function preservation rate (H-B grade I-II) postoperatively. We believe that this satisfactory preservation rate outcome of 68.8% may be related to the use of an endoscopic-assisted technique in our case series. After opening the internal auditory canal, the residual tumor in the internal auditory canal was observed under the endoscope. The endoscope’s clear wide-angle view had a superior advantage over the microscope’s viewing field, thus nerves passing along the tumor capsule can be identified more clearly. Therefore, in patients with preserved hearing functions, both the vestibular and the facial nerve could be identified and preserved throughout the removal of the internal auditory canal tumor. The utilization of endoscopic-assisted microsurgery can avoid nerve damage caused by the blind scraping of nerve dissections under the simple microscope field, and increase the probability of preserving facial and acoustic nerve function after surgery. Hearing loss occurs the most in patients undergoing VS surgery; thus, hearing preservation represents a difficult challenge. Many authors pointed out a correlation between tumor size and hearing preservation, suggesting that larger tumors are associated with poorer postoperative outcomes due to the resultant surgical complexity of these larger tumors (20–22). Good tonal audiometry can be achieved postoperatively with satisfactory results in selected patients with small tumors and good preoperative hearing. Postoperative hearing loss is thought to be related to the intraoperative direct impairment of the cochlear nerve and/or ischemia of nourishing vessels, which leads to cochlear nerve dysfunction (23). According to the results of our study, only 2 of the 16 patients had the same hearing preservation after surgery, 11 patients had varying degrees of hearing loss after the operation, and 6 patients had complete hearing loss.

In the process of microscopic surgery, to expand the surgical field and completely remove the tumor and particularly to expose the lesions growing deep into the midline, it is often necessary to remove more bone, or even free the facial nerve, which may involve other structures such as jugular foramen, pontine triangle, internal carotid artery, high jugular bulb and other important structures that have a high surgical risk when we remove the bone of the internal auditory canal. The damage to the bony labyrinth and semicircular canal when the bony structure of the posterolateral internal auditory canal is removed is also one of the important factors affecting hearing preservation during surgery. Kouhi et al (24) studied the hearing preservation rate of 30 patients after VS surgery and concluded the impossibility of exposing the entire length from the lip to the bottom of the internal auditory canal without causing damage to the posterior semicircular canal. However, utilizing an endoscopic-assisted technique can reduce the extent of bony resection and help prevent intraoperative hearing loss. Ammirati et al. (25) reported that the most commonly damaged structures were: the common peduncle (52%), the posterior semicircular canal (23%), the vestibule (21%), and the superior semicircular canal (4%). Lui et al. (26) studied the safe resection area between the external lip of the internal auditory canal and the posterior semicircle in 120 patients. The maximum safe resection range of the posterior lip of the internal auditory canal was 7-9 mm, and the distance between the posterior superior wall of the internal auditory canal to the anterior edge of the jugular foramen was (3.94 ± 1.75) mm, moreover, the study showed that the length of the posterolateral wall of the internal auditory canal was 9.7 +/- 1.6 mm. Pillai et al. (27) reported the drilling angle (the angle between the drill bit and the posterolateral side of the petrous bone) is 43.3 +/- 6.0 degrees, and the length of the internal auditory canal posterior wall that can be ground without violating the integrity of the labyrinth is 7.2 +/- 0.9 mm. All these results showed that the extent of the safe resection zone varied widely, and the viewing angle and size of the craniotomy also greatly impacted the limit of exposure. Therefore, endoscopic assistance could further improve the intraoperative field of vision and illumination. According to our statistics, the use of the endoscopic-assisted technique in this group of cases showed that the grinding length of the posterior wall of the internal auditory canal in 16 patients was 5.5+/- 0.6 mm, the drilling angle was 42.3 +/- 5.8 degrees (axial plane), and the width of the internal auditory canal was 4.5 +/- 0.5 mm (sagittal plane) and all tumors were completely removed. Postoperative CT scans show no damage to the semicircular canal of all patients, which is superior to the data reported in the previous literature (28, 29). Our experience is that in the case of reducing the exposed length of the posterior of internal auditory canal walls, the width of the internal auditory canal bone resection can be appropriately increased by utilizing the good viewing field and angle of the endoscopy and the ductility of the microdissection device. All that was done to achieve a safer separation of the tumor from the facial auditory nerve under a good field of view as well as to reduce the probability of postoperative neurological damage.

At the same time, the use of the endoscopic-assisted technique can also reduce the incidence of postoperative complications of VS. The most common complications after VS surgery are cerebrospinal fluid leakage, wound infection, as well as postoperative delayed bleeding. The literature reports that the risk of postoperative CSF leakage is 0-17%, mainly caused by the opening of the mastoid air cells when the bone of the internal auditory canal is removed, or for injuries of the bone labyrinth (14, 29, 31). This can be favored by the blind view of the microscope, which failed to seal the open-air cell tightly with postoperative CSF leakage and possible meningitis (14, 29, 31). The 30° endoscope is convenient for close-up and wide-angle observation of tumor resection in the internal auditory canal, which can reduce the extent of opening of the posterior and lateral walls of the internal auditory canal, and at the same time, the opened mastoid air cells can be tightly sealed under a good viewing angle to reduce the risk of CSF leakage. Due to the large size of large acoustic neuromas, their tumors are often embedded in the medial side of the cerebellum and brainstem, they are also closely adhered to the trigeminal nerve, posterior cranial nerve, superior cerebellar artery, anterior inferior cerebellar artery, and petrosal vein. Endoscopic-assisted surgery expands the surgical field, with additional advantages such as overcoming the blind angle of some anatomical areas when only observed under the microscope, minimizing surgical traction, and improving visualization of the neurovascular structures (32, 33).

In addition, with the endoscopic-assisted microsurgery technique, none of the 16 patients in this group had cerebrospinal fluid leakage after surgery, and no serious complications such as death and disability occurred.

In the actual surgical operation, the use of endoscopic-assisted technique involves long-term training and accumulation of operating experience.

Unlike the operation under the 3D image under the microscope, endoscopic images are 2D, thus requiring specific surgical skills to achieve good surgical results. With the development of technology, the 3D endoscope has been gradually used in clinical practice, which will greatly help the application of neuroendoscope (34). At the same time, because this study is a retrospective analysis of the treatment of large VS, the number of cases is relatively small, and the individual differences of patients will inevitably lead to sample bias, which also requires us to further improve the work in the future.

## 5 Conclusion

With the rapid development of neurosurgery technologies, VS’s better intraoperative and postoperative treatment goals are now more attainable. According to the experience of this group of cases, we believe that endoscopic-assisted microsurgery can give neurosurgeons a large and panoramic surgical field to remove VS, help to increase the rate of preserving the facial and acoustic nerves function, and reduce postoperative complications.”

## Data availability statement

The original contributions presented in the study are included in the article/supplementary material. Further inquiries can be directed to the corresponding author.

## Ethics statement

The studies involving human participants were reviewed and approved by the ethics committee of Renmin Hospital of Wuhan University. Written informed consent for participation was not required for this study in accordance with the national legislation and the institutional requirements.

## Author contributions

ZJ and LD supervised the project. Material preparation, data collection, and analysis were performed by XX, and the first draft of the manuscript was written by ZY and LD. All authors contributed to the article and approved the submitted version.
